# Antioxidant potential of wine polyphenols on hematological indices and apical periodontitis in male rats

**DOI:** 10.1590/1678-7757-2025-0229

**Published:** 2025-08-18

**Authors:** Romulo de Oliveira SALES-JUNIOR, Julissa Denisse Arguello ALVARADO, Bharbara de Moura PEREIRA, Rafaela RICCI, Nathália Evelyn da Silva MACHADO, Murilo Catelani FERRAZ, Rayara Nogueira de FREITAS, Renan Jose BARZOTTI, Antonio Hernandes CHAVES-NETO, Luciano Tavares Ângelo CINTRA, João Eduardo GOMES-FILHO

**Affiliations:** 1 Universidade Estadual Paulista Faculdade de Odontologia Araçatuba SP Brasil Universidade Estadual Paulista (UNESP), Faculdade de Odontologia, Araçatuba, SP, Brasil.; 2 Universidade Estadual Paulista Faculdade de Medicina Veterinária Araçatuba SP Brasil Universidade Estadual Paulista (UNESP), Faculdade de Medicina Veterinária, Araçatuba, SP, Brasil.

**Keywords:** Apical periodontitis, Dealcoholized Red Wine, Blood, Oxidative stress, Rats

## Abstract

**Objective:**

To investigate the effects of dealcoholized red wine supplementation on blood cells and the redox state in male rats with established apical periodontitis (AP).

**Methodology:**

Thirty-two male Wistar rats were assigned to one of four groups: control (C), dealcoholized red wine (DRW), red wine (RW), and alcohol (AL). AP was induced, and supplementation was administered for 30 days, starting 30 days after AP induction. At the end of the 60th day, the maxillae were removed for AP radiographic analysis and blood was collected for blood cell and redox state analysis. Statistical tests were applied (p<0.05).

**Results:**

The C and DRW groups showed higher weight gain percentages (p<0.05). The DRW and AL groups exhibited the smallest and the largest periapical lesion areas, respectively (p<0.05). The RW and DRW groups showed similar red blood cell parameters to the C group but different from the AL group (p<0.05). Lymphocyte counts were smaller in the DRW and RW groups compared to the AL and C groups (p<0.05), and the neutrophil count was lower in the AL group (p<0.05). No significant differences were found in monocytes and in lipid and protein oxidative damage. Superoxide dismutase activity was lower in the AL group (p<0.05). The DRW group presented a higher glutathione concentration compared to the RW and AL groups (p<0.05).

**Conclusion:**

DRW reduced periapical lesion size and altered the blood profile by reducing the lymphocyte count and increasing the concentration of endogenous antioxidants such as GSH in male rats with established AP.

## Graphical abstract

**Figure:**
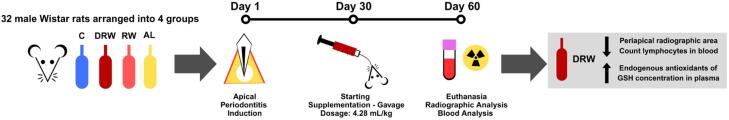

## Introduction

Apical periodontitis (AP) is an inflammatory disease that affects periapical tissues in response to a microbial infection of the root canal system, which can increase inflammation and bone resorption as the infection progressess.^1^ The immune response of the body occurs adaptively via the signaling of defense cells and inflammatory mediators in the bloodstream. Endodontic medicine recognizes AP as an aggravating factor of systemic health.^2^ Infectious metastasis from microbial compounds and inflammatory mediators in the root canal can affect systemic health and alter leukocytes, such as neutrophils and lymphocytes, due to their role in endodontic infection-related inflammation.^3^

Inflammatory mediators are activated by reactive oxygen species (ROS)—highly reactive molecules that help eliminate microorganisms, but can also affect human cells and cause structural damage.^4^ The body produces antioxidants to reduce oxidation, promoting balance;^4^ however, in cases of chronic inflammation such as AP, excessive ROS production can surpass antioxidant capacity, leading to oxidative stress.^5^

Beverages with bioactive compounds, including red wine (RW), have been linked to disease prevention.^6^ RW contains flavonoid and non-flavonoid phenolic compounds, which contribute to vascular protection, bone remodeling, and have antioxidant, anti-inflammatory, and antibacterial effects.^7^ Despite the reported health benefits, alcohol consumption is not universally recommended, due to genetic variability, lifestyle factors, and links to chronic diseases.^8^ Alcohol can impair immune responses, alter cytokine production, and promote microbial proliferation, potentially worsening AP.^9^

A viable alternative is dealcoholized red wine (DRW), which retains the beneficial phenolic compounds of RW without the adverse effects of alcohol, following advances in winemaking that enable alcohol reduction while preserving bioactive components.^10^ DRW contains up to 24% phenolic compounds and has been linked to cardiovascular protection, reduced insulin resistance, lower blood pressure, and high antioxidant capacity.^11,12^ Endodontic studies have shown that DRW, RW, and polyphenols demonstrate the potential to modulate AP by reducing inflammation and bone resorption, whereas alcohol itself can exacerbate these effects.^13–15^

To date, there is no scientific evidence linking the supplementation of polyphenols in DRW as a beneficial supplement for the blood parameters of male rats with established AP. Therefore, this study analyzed the effect of supplementation of DRW on blood cells and the blood plasma redox state in male rats with established AP. The null hypothesis was that DRW supplementation does not alter the blood profile regarding blood cells and the blood plasma redox state in male rats with established AP.

## Methodology

### Ethics statement

This study and its experimental protocols were approved by the Institutional Ethics Committee on Animal Use (2021–2022) of the São Paulo State University (UNESP), Brazil, following National Centre for the Replacement, Refinement and Reduction of Animals in Research (NC3Rs) recommendations.

### Sample calculation

The sample size was calculated on the basis of previous studies with a similar methodology, informed by the parameters used, with a 5% alpha error and a 95% power, indicating that a minimum of seven animals were required per group.^3,13–16^ Due to the long duration of the study and the risk of complications, one extra animal was added per group, totaling eight rats per group.

### Animals

Thirty-two male Wistar rats (*Rattus norvegicus albinus)*, three months old, weighing between 250 and 350 g, were used. The animals were kept in isolators at 22°C, with 70% humidity and light control (12h light and 12h dark), and offered solid food (Labina-Purina^®^, Paulinia, Brazil) and water *ad libitum.* The animals were arranged into four groups (n=8), as follows: control (C) – rats with AP; dealcoholized red wine (DRW) – rats with AP and diet supplemented with DRW; red wine (RW) – rats with AP and diet supplemented with RW; alcohol (AL) – rats with AP and diet supplemented with a 12.5% alcoholic solution. Animals were randomly assigned to groups based on body weight to ensure balanced baseline conditions for dietary intervention.^17^ The experiment lasted 60 days, with supplementation by gavage starting 30 days after AP onset.

### Induction of AP

On the first day of the experimental protocol, all groups underwent general intramuscular anesthesia with a combination of 10 mg/kg of 2% xylazine (Ceva Saúde Animal, São Paulo, Brazil) and 80 mg/kg of 10% ketamine hydrochloride (Ceva Saúde Animal, São Paulo, Brazil) for AP induction. This procedure consisted of exposing the pulp via a coronal opening of the first and second maxillary and mandibular right molars using a long neck Maillefer drill (Dentsply Sirona, Charlotte, North Carolina, USA) with a 0.5 mm diameter, standardizing the pulp exposures.^1,3,13–16,18,19^ Four teeth were selected to develop AP, as this was deemed the optimal number to induce systemic effects related to the disease.^3,18^ To induce AP, teeth remained exposed to the oral environment for 30 days without intervention.^15^

### Weight and supplemental diet

Drinks were administered by gavage on the 31st day after pulp exposure. The daily dose for all groups was 4.28 mL of red wine/kg of animal body weight.^13–15^ This dose corresponds to 300 mL/day of RW for a 70 kg human, characterized as a beneficial dose.^13,15^ The animals were weighed daily for the calculation of diet dosage and weight gain was recorded as a percentage of the initial and final weights.^14^ The C group received sterilized water as a sham intervention, while red wine and dealcoholized red wine were administered to the RW and DRW groups (Vinho, Bento Gonçalves, RS, Brazil), respectively. The AL group received a 12.5% balanced solution to simulate the effect of alcohol, as alcohol concentrations in wine can vary between 9 and 15%. This concentration has been previously used in studies comparing the presence of alcohol in wine.^13–16^

### Collection and storage of maxilla and blood samples

After 60 days of pulp exposure, the rats were anesthetized intramuscularly and a cardiac puncture was performed to collect 5 mL of blood in vacuum microtubes containing ethylenediamine tetra acetic acid (Weihai Sunway Medical Technology Co., Lt, China) homogenate by inversion.^3^ Then, 2 mL were immediately transferred to a technician for processing and determination of cellular parameters.^3^ The remaining blood samples were centrifuged immediately after collection at 10,000 x g for 10 min at 4°C using an Eppendorf R 5810 centrifuge (Eppendorf, Hamburg, Germany). Plasma (200 μL) was used to determine redox state parameters. Finally, the animals were sacrificed with an overdose of 150 mg/kg sodium thiopental (Thiopentax, São Paulo, Brazil) anesthetic solution and the right maxilla was removed for radiographic analysis.^3^

### Radiographic analysis of AP in maxilla

After maxilla removal, soft tissues were cleaned and dissected to confirm AP. Digital periapical radiographs were taken at an angle perpendicular to the vestibular surface of the first molars.^20^ A Spectro 70X device (Dabi Atlante; Ribeirão Preto, Brazil) set to 70 kVp and 10 mA was used, with an exposure time of 0.8 seconds, a focal distance of 20 cm, and a digital sensor (Acuity direct x-ray photon detection model 005-000124). After obtaining the images, the ImageJ program (version 1.54a; National Institute of Health) was used to delineate the borders of the radiographic lesions in the mesial root canals. The total area of the lesions was calculated in square millimeters (mm²), as previously described.^20^ All radiographs were coded and analyzed by a blind author.

### Analysis of blood cell parameters

The samples were initially coded in order to perform the analysis blindly. They were then analyzed by a technician using an automatic hematology analyzer (BC 2800 Vet; Shenzhen Mindray Animal Medical Technology Co., China) for the quantification of hemoglobin, erythrocytes, mean corpuscular volume, hematocrit, neutrophils, lymphocytes, and monocytes.^3^

### Blood plasma redox state parameters analysis

Biochemical assays were performed using spectrophotometric methods with a microplate reader (PowerWave 340, BioTek, USA) and a UV-Vis spectrophotometer (UV-Visible Spectrophotometer Genesys 50, Thermo Fisher Scientific). All measurements were conducted in triplicate, and results were normalized to total protein content. Protein quantification was performed using the Hartree-Lowry method, with bovine serum albumin (CAS Number: 9048-46-8, Sigma-Aldrich, Germany/USA) as the standard.^21^

Oxidative damage was assessed by lipid peroxidation and protein carbonylation. Lipid peroxidation was assessed using the thiobarbituric acid reactive species (TBARS) method^22^ by shaking the sample with 15% (w/v) trichloroacetic acid (CAS Number: 76-03-9, Sigma-Aldrich, Germany/USA), 0.67% (w/v) thiobarbituric acid (CAS Number: 504-17-6, Sigma-Aldrich, Germany/USA), and 0.25 mol/L HCl (CAS Number: 7647-01-0, Sigma-Aldrich, Germany/USA) with incubation at 100 °C for 45 minutes. After cooling, the absorbance was read at 532 nm, using a blank sample as a reference. Aldehyde levels were calculated using the extinction coefficient (ε_532_ = 1.56 × 10^5^ M^−1^ cm^−1^). Protein carbonylation was quantified using the alkaline 2,4-dinitrophenylhydrazine (CAS Number: 119-26-6, Sigma-Aldrich, Germany/USA) method, with absorbance at 450 nm, and carbonyl content was calculated using the molar absorption coefficient (ε_450_ = 22.308 M^−1^ cm^−1^).^23^

Non-enzymatic antioxidant defenses were assessed by measuring total antioxidant capacity and glutathione (GSH) concentrations. Total antioxidant capacity was determined using the ferric reducing antioxidant power (FRAP)^24^ assay, with results obtained from a standard curve generated with varying concentrations of ferrous sulfate heptahydrate solution (CAS Number: 7782-63-0, Sigma-Aldrich, Germany/USA). GSH concentrations were measured via spectrophotometry using 5,5’-dithiobis-(2-nitrobenzoate) (CAS Number: 69-78-3, Sigma-Aldrich, Germany/USA) and quantified based on its molar extinction coefficient (ε_420_ = 1.36 × 10^4^ M^−1^ cm^−1^).^25^

Finally, the enzymatic antioxidant defense assay for superoxide dismutase (SOD) activity was assessed using spectrophotometry at 420 nm, measuring the inhibition of pyrogallol (CAS Number: 87-66-1, Sigma-Aldrich, Germany/USA) autoxidation in 50 mmol/L Tris-HCl buffer (CAS Number: 77-86-1, Sigma-Aldrich, Germany/USA, pH 8.2) containing 1 mmol/L diethylenetriaminepentaacetic acid (CAS Number: 67-43-6, Sigma-Aldrich, Germany/USA). One unit of SOD was defined as the amount of enzyme that inhibits 50% of autoxidation.^26^

### Statistical analysis

Data were analyzed using SigmaPlot 14.0™ (Chicago, IL, USA). Normality was assessed using the Shapiro-Wilk test, and variance homogeneity was verified using the Brown-Forsythe equal variance test. Since all variables met these requirements, they were treated as parametric. One-way analysis of variance (ANOVA) followed by Tukey *post hoc* test was applied. A 5% significance level (p<0.05) was adopted.

## Results

### Body weight gain

**Figure 1:**
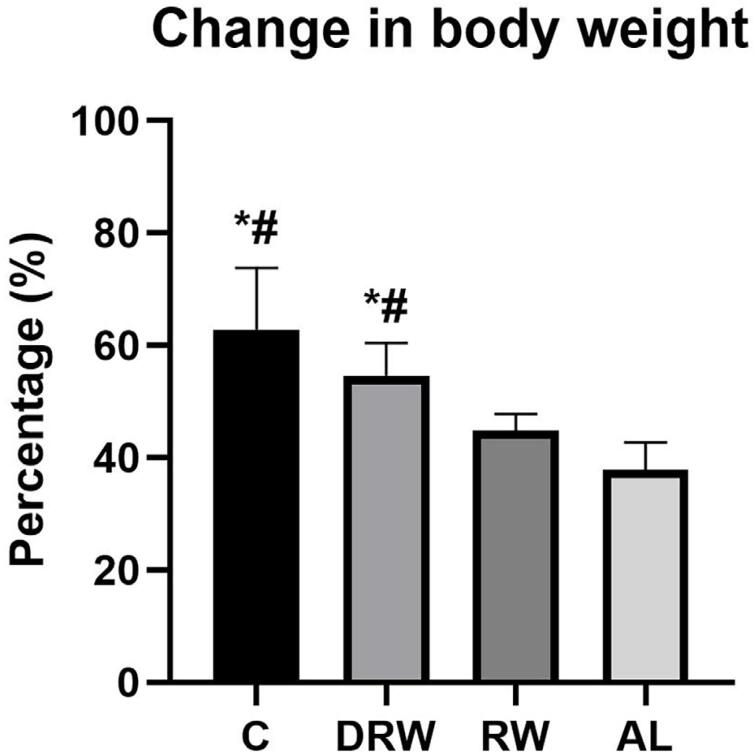
The bar graph shows the mean and standard deviation values of percentage increases in weight. Variables met normality (Shapiro-Wilk) and equal variance (Brown-Forsythe) assumptions. One-way ANOVA with Tukey *post hoc* test was applied (n=8, p<0.05). Symbols indicate statistical differences: *p<0.05 vs. RW; # p<0.05 vs. AL.

All animals gained weight during the experimental period, with mean and standard deviation values of 62.75%±11.04 for the C group, 54.63±5.82 for the DRW group, 44.87%±2.09 for the RW group, and 37.75%±4.92 for the AL group (Figure 1). The mean body weight gain values showed that the C and DRW groups gained significantly more weight than the RW and AL groups (p<0.05).

### AP radiographic analysis

**Figure 2:**
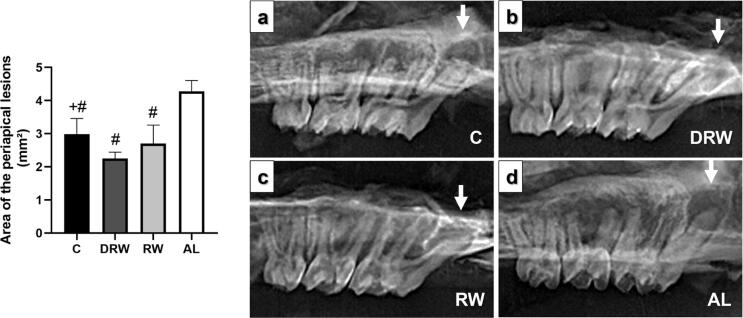
Bar graph of periapical lesion area (mm²) with mean and standard deviation for each group. Variables met normality (Shapiro-Wilk) and equal variance (Brown-Forsythe) assumptions. One-way ANOVA with Tukey *post hoc* test was applied (n=8, p<0.05). Symbols indicate statistical differences: +p<0.05 vs DRW; # p<0.05 vs. AL. Representative radiographic images of the mesial root canals periapical areas of the maxillary first molars. All groups presented radiolucent areas (white arrows).

Figure 2 presents the radiographic analysis graph with representative images. All groups demonstrated an increase in radiolucent areas in the periapical region of the mesial root canals caused by AP. The AP radiographic area (mm^2^) was statistically smaller in the DRW group (2.2±0.19) when compared to the C group (2.9±0.47) and the AL group (4.2±0.33) (p<0.05). There was no difference between the DRW and RW groups (2.7±0.56). The AP radiographic area in the AL group was statistically larger than the other groups (p<0.05).

### Analysis of blood cell parameters

Supplementation affected blood cell parameters (Table 1). In summary, the analysis of the results for hemoglobin, erythrocytes, hematocrit, and mean corpuscular volume showed significantly higher quantitative values in the AL group than in the C, RW, and DRW groups (p<0.05). The percentage of lymphocytes was higher in the AL group (p<0.05) than in all other groups, and in the C group than in the DRW and RW groups (p<0.05). The neutrophil count was lower in the AL group (p<0.05) compared to the other groups. The monocyte count was similar between all groups (p>0.05).

**Table 1:** Mean and standard deviation values of blood cell parameters.

	Groups (Mean ±SD)				
Hematological parameters	Reference values	C	DRW	RW	AL
Hemoglobin (g/dL)	13–15	14.18±0.50a	13.67±0.41^a^	14.50±0.39a^a^	16.17±1.00^b^
Erythrocytes (x10^6^/μL)	6–8	7.99±0.35^a^	7.93±0.35^a^	7.83±0.24^a^	9.23±0.54^b^
Mean corpuscular volume (fL)	48-54	49.91±2.13^a^	49.81±1.70^a^	51.61±1.04^a^	62.24±2.02^b^
Hematocrit (%)	42–49	40.37±1.84^a^	40.00±1.77^a^	40.75±1.66^a^	57.00±2.20^b^
Neutrophils (%)	6–22	20.75±2.43^b^	21.87±0.99^b^	20.37±2.32^b^	14.25±2.31^a^
Lymphocyte (%)	69–86	76.00±2.56^b^	71.00±1.30^a^	71.25±1.48^a^	83.37±2.20^c^
Monocytes (%)	3–10	3.62±0.74^a^	3.12±0.35^a^	3.25±0.46^a^	3.75±0.46^a^

Values are presented as mean ± standard deviation. One-way analysis of variance (ANOVA) followed by Tukey test (n=8). Different lowercase letters in the same line show statistical differences (p<0.05) between groups. Reference values^27^

### Blood plasma redox state parameters analysis

**Figure 3:**
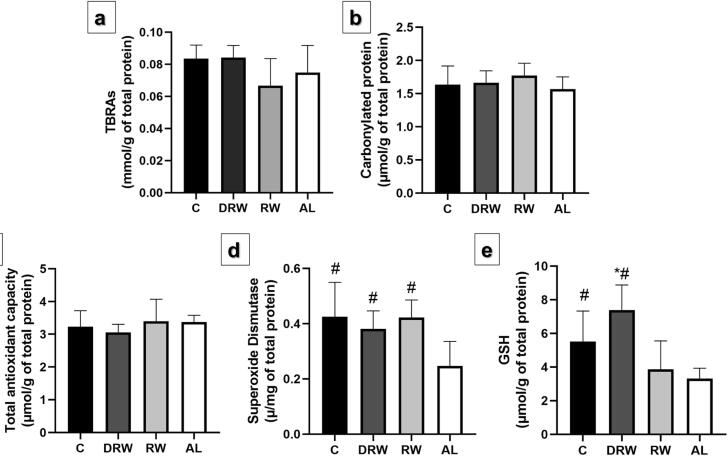
Bar graphs showing the effects of DRW, RW, and AL on plasma redox state parameters in rats with established AP. The analysis included oxidative damage—TBARs (A) and protein carbonyl (B); non-enzymatic antioxidant defense—total antioxidant capacity (C); and GSH (D); and enzymatic antioxidant defense—SOD (F). Data are expressed as mean ± standard. Variables met normality (Shapiro-Wilk) and equal variance (Brown-Forsythe) assumptions. One-way ANOVA with Tukey *post hoc* test was applied (n=8, p<0.05). Symbols indicate statistical differences (p<0.05): *p<0.05 vs. RW; # p<0.05 vs. AL.

Supplementation in the groups led to changes in blood redox state parameters (Figure 3). In summary, there was no lipid oxidative damage or protein oxidative damage measured by TBARs and protein carbonyl concentrations. Although total antioxidant capacity was similar between groups, there was a reduction in enzymatic oxidant defense for SOD concentration in the AL group compared to the other groups (p<0.05). In addition, an increase was observed in non-enzymatic oxidant defense for GSH in the DRW group compared to the RW group (p<0.05) and a reduction in the AL group compared to the DRW and C groups (p<0.05).

## Discussion

To the best of our knowledge, this is the first study to analyze the influence of DRW on the blood profile and redox status of male rats with established AP. The findings demonstrate that DRW supplementation altered the blood profile by reducing lymphocytes, increasing the concentration of endogenous antioxidants such as GSH, and simultaneously reducing the periapical lesion size in male rats. These results suggest that systemic changes induced by DRW may play a role in modulating the inflammatory response and bone resorption in AP, leading to the rejection of the null hypothesis.

AP was induced by exposure of the pulp to oral microorganisms, using methodologies from previous studies, leading to pulp necrosis and periapical tissue damage.^1,3,13–16,18,19^ The gavage method was adopted to administer beverages, with a beneficial dose based on previous studies that used body weight to standardize the dosage.^13–15^

The individual dose was determined based on the daily weight of each rat, enabling an analysis of the percentage increase in body weight. The groups receiving RW and AL supplementation presented the lowest mean weight gain, compared to C and DRW. This outcome may be attributed to the high caloric content of red wine and alcohol, which has been reported to induce satiety in animals, potentially reducing overall food intake.^28^ However, ethanol intake is known to cause fatty malnutrition and impair nutrient absorption,^14,29^ which could further explain the reduced weight gain observed in the RW and AL groups. These findings were previously reported with DRW, RW and AL supplementation.^14^

The experimental AP model adopted in this study was validated by the increased radiolucent areas in the periapical region of the mesial root canals, consistent with findings from other studies.^20^ The DRW group showed the smallest lesion areas, as opposed to AL, which had the largest lesion areas, consistent with previous studies linking DRW to reduced bone resorption and AL to increased bone resorption.^13–15^ Studies report that polyphenols present in wine can modulate the host osteoimmune inflammatory response by reducing inflammatory cytokines related to osteoclast maturation, such as nuclear factor kappa-B.^13–15^ Ethanol can promote osteoclastogenesis via the receptor activator of nuclear factor kappa-B ligand, linked to pro-inflammatory cytokines, which are released upon exposure.^16^

The comparative analysis of blood cells was based on established hematological reference ranges.^27^ The RW and DRW groups showed hemoglobin and erythrocyte counts, mean corpuscular volume (MCV) and hematocrit similar to the C group, likely due to the antioxidant properties of polyphenols, which counteract the oxidative damage caused by ROS.^12,30^ Interestingly, the AL group showed elevated levels of these parameters. Although anemia is frequently associated with chronic alcohol consumption, this relationship is influenced by dose, prolonged exposure over several years, and systemic condition.^8,31,32^ The increase in MCV, as observed in this study, is a known biomarker of alcohol exposure and may reflect oxidative damage to erythrocyte membranes.^12,33^ Alcohol can also increase ferritin and iron absorption, contributing to higher hemoglobin levels.^34^ Thus, while these findings differ from what is typically reported, they align with literature describing the complex hematological effects of alcohol.^31,32^ The absence of such alterations in the DRW group suggests an effect in maintaining hematological balance. Further studies are needed to clarify these mechanisms.

The DRW and RW groups showed lower lymphocyte counts, likely due to the antioxidant properties of polyphenols, which protect immune cells from oxidative damage.^12^ These findings align with previous studies reporting no adverse effects of RW and DRW on immune cell function in healthy individuals,^35^ and a reduction in lymphocyte count in wine-supplemented rats.^36^ In contrast, the AL group exhibited a significant increase in lymphocytes, particularly T lymphocytes, which play a key role in the chronic phase of AP.^37^ This may be attributed to the stimulatory effect of alcohol on naive lymphocyte maturation into memory T cells, enhancing their sensitivity and pro-inflammatory cytokine production.^38^

Moreover, monocyte count was similar among the groups, likely due to the chronic nature of AP.^39^ On the other hand, neutrophil counts were higher in the DRW and RW groups, possibly due to the protective effect of polyphenols against oxidative stress, which helps maintain immune function.^35^ In contrast, the AL group showed a decrease in neutrophil concentration. Although alcohol may promote neutrophil recruitment in innate immunity, it appears to have a suppressive effect on adaptive immunity, potentially increasing susceptibility to bacterial infections.^40^

In this study, supplementation led to significant changes in blood redox status parameters, particularly highlighting the antioxidant benefits of DRW. The results indicate an increase in GSH concentration in the DRW group compared to the RW and AL groups, while the AL group showed a reduction in SOD activity. The increase in GSH in the DRW group compared to RW suggests that wine polyphenols favor non-enzymatic endogenous antioxidant defense without the interference of alcohol. Phenolic compounds can positively contribute to increasing plasma antioxidant capacity via GSH concentrations and stimulating the activity of glutathione reductase and glucose-6-phosphate dehydrogenase.^41^ A reduction in GSH reflects alcohol-related depletion, increased oxidative stress, and impaired immune response.^32,42^ The increased lymphocytes and decreased neutrophils in the AL group support oxidative imbalance in immune dysregulation.

Despite these influences on antioxidant defense mechanisms, no significant oxidative damage to lipids or proteins was observed among the groups. TBARs levels and carbonyl protein concentrations remained similar, indicating that none of the interventions induced excessive oxidative stress. Unlike studies with shorter AP durations (≤30 days), which reported increased TBARS levels,^18,19^ the absence of significant differences in our model may reflect a compensatory redox response during a more chronic disease phase. This is supported by the unaltered total antioxidant capacity, suggesting that oxidative stress may be mitigated via alternative pathways over time. Further studies at different times are warranted to clarify this dynamic. However, the reduction in enzymatic and non-enzymatic antioxidant defense in the AL group reinforces that alcohol compromises redox homeostasis, causing an imbalance. The decrease in SOD reinforces the possible detrimental effects of alcohol on enzymatic antioxidant defense with increased production of ROS during ethanol metabolism, which overloads antioxidant mechanisms and leads to depletion of protective enzymes.^43^

While previous studies with red wine polyphenols in apical periodontitis focused on local effects,^14,15^ our study extends the knowledge to the bidirectional interaction between systemic oxidative-inflammatory status and the periapical bone lesions in the context of chronic oral infections. Although the clinical management of apical periodontitis typically involves infection removal via root canal therapy, these findings contribute to understanding the potential of dietary interventions. In this context, polyphenols from DRW may serve as an adjunctive systemic approach to support the host response, providing insights for future clinical research on the therapeutic potential of its polyphenols. However, due to limitations such as the use of animal models, the inclusion of only male rats, wine dosage, and the complexity of treating apical periodontitis for its resolution, the results cannot be directly extrapolated to humans. These factors highlight the need for future studies that take all these limitations into account, including confirming whether the findings are consistent across genders.

## Conclusion

The consumption of DRW led to a smaller periapical radiographic area and altered the blood profile by reducing lymphocytes and increasing the concentration of endogenous antioxidants such as GSH in male rats with established AP. This study highlights the potential of systemic administration of polyphenols from DRW to help maintain immuno-redox balance in the context of oral inflammatory diseases.
